# Biological invasion with a porous medium type diffusion in a heterogeneous space

**DOI:** 10.1007/s00285-024-02124-6

**Published:** 2024-07-21

**Authors:** Hyunjoon Park, Yong-Jung Kim

**Affiliations:** 1 Meiji Institute for Advanced Study of Mathematical Sciences, Meiji University, Nakano, Nakano-ku, Tokyo, 164-8525 Japan; 2 Department of Mathematical Sciences, KAIST, 291 Daehak-ro, Yuseong-gu, Daejeon, 34141 Korea

**Keywords:** Porous medium diffusion, Biological invasion, Hyperbolic singular limit, Interface Motion, Traveling wave, 35B25, 35K57, 82C24

## Abstract

The knowledge of traveling wave solutions is the main tool in the study of wave propagation. However, in a spatially heterogeneous environment, traveling wave solutions do not exist, and a different approach is needed. In this paper, we study the generation and the propagation of hyperbolic scale singular limits of a KPP-type reaction–diffusion equation when the carrying capacity is spatially heterogeneous and the diffusion is of a porous medium equation type. We show that the interface propagation speed varies according to the carrying capacity.

## Introduction

The purpose of the paper is to show how the invasion speed of a KPP-type reaction–diffusion equation varies under spatial heterogeneity when a porous medium equation-type nonlinear diffusion is taken. More specifically, we first show how a sharp interface of the solution to an initial value problem,

1.1 $$\begin{aligned} {\left\{ \begin{array}{ll} U_t(x,t) =\varepsilon \Delta U^{m}+\frac{1}{\varepsilon } U\big (1-\frac{U}{k(x)}\big ),&{} (x,t) \in D \times \mathbb {R}^+ ,\\ \frac{\partial U}{\partial \nu } =0,&{} (x,t) \in \partial D \times \mathbb {R}^+,\\ U(x,0) = U_0(x)\in [0,k(x)], &{} x \in D, \end{array}\right. } \end{aligned}$$

is generated when $\varepsilon >0$ is small. Then, we obtain the propagation speed of the interface after taking the singular limit as $\varepsilon \rightarrow 0$. The solution *U*(*x*, *t*) is the population density of a biological species, the domain $D\subset {\mathbb {R}}^N$ is smooth and bounded, and the vector $\nu$ is the outward unit normal vector on the boundary of the domain. In this model, we take the nonlinear diffusion with a constant exponent $m\ge 2$. The spatial heterogeneity is placed in the carrying capacity, $k=k(x)>0$, which satisfies

1.2 $$\begin{aligned} k\in C^2(D),\ \ c_k \le k \text { and }\ k + |\nabla k| + |\Delta k| \le C_k \end{aligned}$$

for some positive constants $C_k, c_k > 0$.

**Fig. 1:**
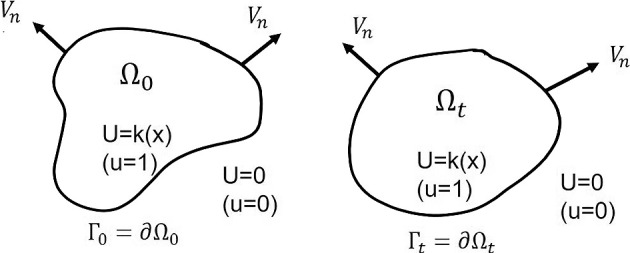
A diagram for interface propagation of the singular limit

The problem (1.1) is obtained after a hyperbolic rescaling, $x\rightarrow \varepsilon x$ and $t\rightarrow \varepsilon t$, of a multi-scale problem, where the heterogeneity in *k*(*x*) is not rescaled since it is of a macroscopic scale. Evans and Sougandis (1989, Eq. (1.1)) considered such hyperbolic multi-scale problem for a general heterogeneous reaction function. However, the reaction function in (1.1) does not satisfy their assumptions. Since the wave speed of the problem is invariant under the hyperbolic scaling of the problem, this approach provides the wave propagation speed in a heterogeneous environment. Hilhorst et al. (2008) considered a homogeneous case with $k(x)=1$ and showed that the solution *U*(*x*, *t*) converges to 0 or *k*(*x*) as $\varepsilon \rightarrow 0$ and the interface moves with a constant speed to the normal direction, i.e.,

1.3 $$\begin{aligned} V_n = {c_0} , \end{aligned}$$

where $V_n$ denotes the speed of the propagating interface in the normal direction and ${c_0}>0$ is the minimum wave speed of traveling wave solutions in one space dimension (see Fig. 1). The constant speed ${c_0}$ depends on the nonlinearity $m$, but not on the space dimension $d>0$.

If the coefficients are constant, we can easily compute the propagation speed by changing variables, as in Section 7. In the paper, we show that if $k=k(x)$,

1.4 $$\begin{aligned} V_n = {c_0} k^p(x)\quad \text {for}\quad p:=\frac{m-1}{2}, \end{aligned}$$

where the invasion speed is not constant anymore. There are three interesting observations in this relation. First, even if there is no traveling wave solution in the heterogeneous case, the traveling wave speed ${c_0}$ of the homogeneous case with $k=1$ still plays a key role. Second, if (1.4) holds for the linear diffusion case $m=1$, which is beyond the parameter regime of the paper, the heterogeneity in *k*(*x*) does not make any difference in the invasion speed. It is related to the well-known fact for the linear diffusion case that the first-order term of the reaction decides the invasion speed. In other words, the relation (1.4) says that such a well-known fact is true only for the linear diffusion case, and the wave speed depends on the second-order term for the nonlinear diffusion case.

To obtain the invasion speed in a heterogeneous environment, we first transfer the spatial heterogeneity in the reaction function to the diffusion operator and obtain a reaction function that satisfies the hypotheses of Evans and Sougandis (1989, (1.2)–(1.4)). To do that, we rewrite the equation in terms of the ratio,

$$\begin{aligned} u(x,t):= \frac{U(x,t)}{k(x)}. \end{aligned}$$

This ratio is the number of populations that share a unit amount of resources, where the ratio has been used as a starvation measure in Kim and Kwon (2016). Then, (1.1) becomes

1.5 $$\begin{aligned} {\left\{ \begin{array}{ll} u_t(x,t)=\frac{\varepsilon }{k}\Delta (ku)^{m}+\frac{1}{\varepsilon }u(1-u), &{} (x,t) \in D \times \mathbb {R}^+,\\ \frac{\partial (ku)^m}{\partial \nu }=0, &{}(x,t) \in \partial D \times \mathbb {R}^+,\\ u(x,0) = u_0(x):=\frac{U_0(x)}{k(x)}, &{}x \in D. \end{array}\right. } \end{aligned}$$

We need several assumptions on the initial value. The initial value $u_0$ is uniformly bounded by

1.6 $$\begin{aligned} 0\le u_0(x)\le 1, \end{aligned}$$

has a smooth and simply connected compact support,

1.7 $$\begin{aligned} \Omega _0:=\{x\in D: u_0(x)>0\} \subset \subset D \ \text { and }\ \Gamma _0 := \partial \Omega _0 \in C^{3 + \alpha } \end{aligned}$$

for a $0<\alpha <1$, and has smoothness and boundary steepness,

1.8 $$\begin{aligned} u_0 \in C^2(\overline{\Omega }_0)\cap C(D)\ \text { and }\ \dfrac{\partial u_0}{\partial \nu _0}(x_0)<-C_0\ \text { for }\ x_0 \in \Gamma _0 \end{aligned}$$

for a constant $C_0>0$. The vector $\nu _0$ is the outward unit normal vector on the boundary $\Gamma _0$ of the support of the initial value.

Notice that the support $\Omega _0$ of the initial value is not assumed to be convex since it does not mean much. For a homogeneous problem case, the interface of the solution moves with constant speed (1.3), and the convexity of the solution support is preserved. However, for a heterogeneous case, the corresponding flow is (1.4), and the convexity of the solution support may break (see Fig. 3). Instead of the convexity, the support $\overline{\Omega _0}$ is assumed to be simply connected, and hence, the boundary $\Gamma _0:=\partial \Omega _0$ is a simple loop and divides the domain $D\subset {\mathbb {R}}^d$ into two regions.

## Main results

The main results of the paper are the next two theorems. The first one shows how the interface is created. In the following theorem, we show that for any $t>0$, the limit of the solution $\lim _{\varepsilon \rightarrow 0}u^\varepsilon (x,t)$ is a step function that connects 0 and 1. We call the curve of the discontinuity *interface*. To see the development of the interface for small $\varepsilon >0$, we consider a transition layer, which is a region in which the solution $u^\varepsilon$ takes values between 0 and $1-\eta _g$ for an arbitrarily small $\eta _g>0$.

### Theorem 2.1

(Generation of interface) Let *k*(*x*) satisfy (1.2) and $u_0(x)$ satisfy (1.6)–(1.8). Let $u^\varepsilon (x,t)$ be the solution of (1.5) in a weak sense and let $\eta _g>0$. Then, there exist positive constants $\varepsilon _0$, $M_G$, and $\eta _\varepsilon >0$ for all $\varepsilon \in (0, \varepsilon _0)$ such that $\eta _\varepsilon =\mathcal {O}(\varepsilon ^2)$ as $\varepsilon \rightarrow 0$ and, for $t^\varepsilon := \varepsilon |\ln \varepsilon |$,

2.1 $$\begin{aligned} 0\le & {} u^\varepsilon (x,t) \le 1 {+ \eta _\varepsilon }, \end{aligned}$$

2.2 $$\begin{aligned} u^\varepsilon (x,t^\varepsilon )\ge & {} 1 - \eta _g \quad \text {if} ~~ u_0(x)\ge M_G\varepsilon , \end{aligned}$$

2.3 $$\begin{aligned} u^\varepsilon (x,t^\varepsilon )= & {} 0 \quad \text {if} ~~ dist(x, \Omega _0) \ge M_G \varepsilon . \end{aligned}$$

This theorem provides inner and outer envelopes for the graph of $u^\varepsilon (\cdot ,t^\varepsilon )$. Note that the solution $u^\varepsilon$ is not bounded above by 1 in (2.1) due to the Fokker-Plank type diffusion in (1.5) even if the initial value $u_0$ is. The estimate (2.2), the boundary steepness (1.8), and the equality (2.3) imply that a transition layer of thickness $\mathcal {O}(\varepsilon )$ is developed along the boundary $\Gamma _0$ of the initial support $\Omega _0$ at the moment of $t^\varepsilon =\varepsilon |\ln \varepsilon |$. If the initial value is larger than $M_G\varepsilon$ at a point $x\in \Omega _0$, $u^\varepsilon (x,t^\varepsilon )$ is between $1+\eta _\varepsilon$ and $1-\eta _g$ by (2.1) and (2.2). Due to the assumption (1.8) on the initial value, the transition layer is at the distance of utmost $\mathcal {O}(\varepsilon )$ to the boundary $\Gamma _0$. Equation (2.3) implies that the solution remains equal to zero $u(x,t^\varepsilon )=0$ on the outside of the layer. The outer layer $1+\eta _\varepsilon$ converges to 1 with the second order as $\varepsilon \rightarrow 0$. However, we did not obtain the convergence order for the inner layer $1-\eta _g$. If we want to give a convergence order to the inner layer, we may lose the thickness of the sharp interface of order $\mathcal {O}(\varepsilon )$. Hence, $\eta _g>0$ is fixed but can be arbitrarily small. Therefore, after taking the limit as $\varepsilon \rightarrow 0$, $u^\varepsilon (x,t^\varepsilon )$ converges to a step function, which is 1 for $x\in \Omega _0$ and 0 for $x\notin \Omega _0$. The boundary $\Gamma _0$ is the initial interface of discontinuity of this singular limit.

The second theorem is to show that the interface of the step function $u(x,t):=\lim _{\varepsilon \rightarrow 0}u^\varepsilon (x,t)$ moves according to the relation (1.4). To make the statement explicit, we construct a step function with its interface moving according to (1.4) and then show that $u^\varepsilon (x,t)$ converges to the constructed step function. First, define a collection of interfaces $\Gamma _t$ indexed with the time variable $0\le t\le T$, which satisfy a heterogeneous motion equation,

2.4 $$\begin{aligned} V_n(x) = {c_0} k^p(x) ~ \text {for} ~x\in \Gamma _t, \quad \Gamma _t|_{t = 0} = \Gamma _0, \end{aligned}$$

where $V_n(x)$ is the speed of the moving interface in the outward normal direction at position $x\in \Gamma _t$ and at time $0<t<T$. The coefficient $c_0$ is the traveling wave speed for the homogeneous case with $k=1$. Under the regularity of *k* given in (1.2) and the assumption that the interface $\Gamma _t$ does not touch itself and the boundary $\partial D$, the flow is defined well (see Sect. 3).

Let

$$\begin{aligned} D=\Omega _t\cup \Omega ^c_t, \end{aligned}$$

where the *outer region* $\Omega ^c_t$ is the area bounded by $\Gamma _t$ and $\partial D$, and the *interior region* $\Omega _t$ is the rest which is enclosed by the interface $\Gamma _t$. Note that $\Omega _0$ is the support of the initial value $u(x,0)=u_0(x)$. However, $\Omega _t$ is not the support of *u*(*x*, *t*). It is simply the interior region bounded by the $\Gamma _t$ and $\Gamma _t$ is given by the heterogeneous curvature-flow (2.4). Finally, we define a signed distance function $\tilde{d}(x,t)$;

$$\begin{aligned} {\left\{ \begin{array}{ll} \tilde{d}(x,t) =\ \ dist(x, \Gamma _t)&{}\text {if}~~ x \in \Omega _t,\\ \tilde{d}(x,t) = - dist(x, \Gamma _t) &{}\text {if}~~ x \in \Omega ^c_t. \end{array}\right. } \end{aligned}$$

Note that ${\tilde{d}}(x,t)\le 0$ if *x* is in the outer region.

The second theorem is about the propagation of the interface. Our analysis is valid only before $\Gamma _t$ touches itself or the distance to the boundary $\partial D$ is not too close. The maximum distance between $\Gamma _t$ and $\partial D$ will be $2d_0$, which will be decided in Lemma 3.5.

### Theorem 2.2

(Propagation of interface) Let $\eta _p>0$, $t^\varepsilon := \varepsilon |\ln \varepsilon |$, *k*(*x*) satisfy (1.2), $u_0(x)$ satisfy (1.6)–(1.8), and $u^\varepsilon (x,t)$ be a weak solution of (1.5). Then, there exist a time $T>0$, positive constants $\varepsilon _0$ and $M_P$, and $\eta _\varepsilon >0$ for all $\varepsilon \in (0, \varepsilon _0)$ such that $\eta _\varepsilon =\mathcal {O}(\varepsilon ^2)$ as $\varepsilon \rightarrow 0$ and, for $x\in D$ and $t^\varepsilon<t<T$,

2.5 $$\begin{aligned} {\left\{ \begin{array}{ll} 0 \le u^\varepsilon (x,t) \le 1 {+ \eta _\varepsilon }, &{}\\ u^\varepsilon (x,t) \ge 1 - \eta _p &{}\text { if } \tilde{d}(x,t) \ge \ \ M_P \varepsilon ,\\ u^\varepsilon (x,t) = 0 &{}\text { if } \tilde{d}(x,t) \le - M_P \varepsilon . \end{array}\right. } \end{aligned}$$

The constant $\eta _\varepsilon$ is the one in Theorem 2.1. The results in (2.5) imply that the interface generated at time $t_\varepsilon$ propagates according to the motion equation (2.4) and its thickness is of order $\mathcal {O}(\varepsilon )$. By a modification of the proof of Theorem 2.2, one can prove that

2.6 $$\begin{aligned} u^\varepsilon (x,t) \rightarrow \left\{ \begin{array}{ll} 1,&{}x\in \Omega _t,\\ 0,&{}x\in \Omega _t^c, \end{array} \right. \quad \text {as }\ \varepsilon \rightarrow 0 \end{aligned}$$

(see Remark 1). Furthermore, since the boundary $\partial \Omega _t$ is defined by the relation (2.4), we have obtained the claim of the paper that waves move with the speed as given in (1.4).

Note that the solution in the theorem exists for a time interval [0, *T*]. For a homogeneous case, the solution support is convex if the initial support is convex. Hence, we may construct a solution until the interface touches the domain boundary $\partial D$. If the domain is the whole space ${\mathbb {R}}^d$, the solution exists for all $t>0$. However, the convexity of solution support is not preserved for a heterogeneous case, and the interface $\Gamma _t$ may touch itself. Hence, even if the domain is ${\mathbb {R}}^d$, we may obtain the solution only a finite time.

## Preliminaries

In this section, we define the solution of the problem (1.5) in a weak sense and consider a few preliminaries which are used later in the proof of theorems.

### Weak solution

The solution, the super-solution, and the sub-solution of the perturbed problem (1.5) are defined in a weak sense.

#### Definition 3.1

A function $u:D\times [0,T] \rightarrow {\mathbb {R}}$ is called a super-solution of the singularly perturbed problem (1.5) if

(i) the product $ku \in W^{1,m}({D} \times [0,T])$ and
(ii) for $\phi \in C^1_c(D \times [0,T])$ with $\phi \ge 0$,
  3.1 $$\begin{aligned}{} & {} \displaystyle \int _D u(x,T) \phi (x,T) \ge \int _D u(x,0) \phi (x,0)\nonumber \\{} & {} \displaystyle \quad + \int _0^T \int _D \left( u \phi _t - \varepsilon \nabla (ku)^m\cdot \nabla \frac{\phi }{k} + \frac{1}{\varepsilon } u(1 - u) \phi \right) . \end{aligned}$$

If (3.1) is satisfied with the opposite inequality, *u* is called a sub-solution. The function *u* is called a solution if *u* is a super-solution and a sub-solution at the same time.

Note that the regularity of the product *ku*, which is in $W^{1,m}$, matters due to the diffusion model but not the solution *u* itself. If Fick’s law type diffusion, $u_t=\nabla (k\nabla u)$, is used, the regularity of the solution *u* would be needed. However, we assume *k* is smooth as in (1.2), and hence the solution *u* becomes smooth in the paper. Next, we introduce two basic lemmas. The first lemma is a classical comparison principle (see Vazquez 2007).

#### Lemma 3.2

(Comparison principle) Let $\overline{u}$ and $\underline{u}$ be super- and sub-solutions of (1.5), respectively. If $\overline{u}(x,0) \ge \underline{u}(x,0)$ for all $x \in D$.Then

$$\begin{aligned} \overline{u}(x,t) \ge \underline{u}(x,t),~~~ (x,t) \in D \times [0,T]. \end{aligned}$$

For the estimate in the following sections, we construct smooth super- and sub-solutions. The second lemma is to give sufficient conditions for *u* to be a super- or a sub-solution. Denote

$$\begin{aligned} \mathcal {L} u := u_t - \frac{\varepsilon }{k} \Delta (ku)^m- \frac{1}{\varepsilon }u(1 - u). \end{aligned}$$

#### Lemma 3.3

Let *k* satisfy the conditions in (1.2) and *u* be a differentiable nonnegative function defined on $D \times [0,T]$. Let $D^+_t:=\{x\in D: u(x,t)>0\}$, $n_t$ be the normal outer vector of $D^+_t$ on its boundary $\partial D^+_t$, and the surface $\cup _{t \in [0, T]} \partial D^+_t \times \{t\}\subset D\times [0,T]$ be smooth. Suppose *u* satisfies three conditions; (*i*) $u^m\in C^1(D \times [0,T])$, (*ii*) $\dfrac{\partial (ku)^{m}}{\partial n_t} \ge 0$ on $\partial D^+_t$, and (*iii*) ${\mathcal {L}} u \ge 0$ in $D^+_t$. Then, *u* is a super-solution. If the inequalities in (ii) and (iii) hold in the opposite direction, *u* is a sub-solution.

#### Proof

We will prove the theorem only for a super-solution case. The sub-solution case can be proved similarly. For a nonnegative test function $\phi \in C_c^1(D \times [0,T])$, we have

$$\begin{aligned} \dfrac{d}{dt} \left( \int _{D} u \phi \right)&= \dfrac{d}{dt} \left( \int _{D^+_t} u \phi \right) = \int _{D^+_t}(u \phi _t + u_t \phi ) + \int _{\partial D^+_t} u\phi V_t\\&= \int _{D^+_t}(u \phi _t + u_t \phi ), \end{aligned}$$

where $V_t$ is the speed of the propagating boundary $\partial D^+_t$ in the outward normal direction. The last equality holds since $u = 0$ on $\partial D^+_t$. Integrating both sides over [0, *T*] gives

$$\begin{aligned} \int _0^T \int _{D^+_t} u \phi _t = - \int _0^T \int _{D^+_t} u_t \phi + \int _{D} u(T) \phi (T) - \int _{D} u(0) \phi (0). \end{aligned}$$

Then,

$$\begin{aligned}&\int _{D} u(T) \phi (T) = \int _{D} u_0 \phi (0) + \int _0^T \int _{D^+_t} u \phi _t + \int _0^T \int _{D^+_t} u_t \phi \\&\quad \ge \int _{D} u_0 \phi (0) + \int _0^T \int _{D^+_t} u \phi _t + \int _0^T \int _{D^+_t} \left( \dfrac{\varepsilon }{k} \Delta (ku)^m+ \dfrac{1}{\varepsilon } u(1 - u) \right) \phi \\&\quad = \int _{D} u_0 \phi (0) + \int _0^T \int _{D^+_t} u \phi _t + \int _0^T \left. \varepsilon \dfrac{\phi }{k} \nabla (ku)^m\cdot n_t \right| _{\partial D^+_t}\\&\qquad - \int _0^T \int _{D^+_t} \varepsilon \nabla \dfrac{\phi }{k} \cdot \nabla (ku)^m+ \int _0^T \int _{D^+_t} \dfrac{1}{\varepsilon } u(1 - u) \phi \\&\quad \ge \int _{D} u_0 \phi (0) + \int _0^T \int _{D} \left( u \phi _t - \varepsilon \nabla (ku)^m\cdot \nabla \frac{\phi }{k} + \frac{1}{\varepsilon } u(1 - u) \phi \right) . \end{aligned}$$

Therefore, *u* is a super-solution in the weak sense. $\square$

### Traveling wave solution

The traveling wave solution for a homogeneous case still plays a key role in a heterogeneous case (see Hilhorst et al. 2008). Consider a homogeneous reaction–diffusion equation in one space dimension,

$$\begin{aligned} v_t=(v^m)_{xx}+v(1-v). \end{aligned}$$

Let $v(x,t)=U(x+c_0t)$ be a traveling wave solution with the minimum speed $c_0>0$, where the traveling wave moves from right to left in this setting. There exists a traveling wave solution for each $c\ge c_0$, which is unique up to a translation. The support of the traveling wave solution is the whole real line ${\mathbb {R}}$ if $c>c_0$ (see Atkinson et al. 1981; Biró 2002). However, for the traveling wave solution with the minimum speed $c_0$, the support is a half line $[x_0,\infty )$ for a $x_0\in {\mathbb {R}}$ and we may set $x_0=0$ after a translation. If we denote $z=x+c_0t$, the traveling wave solution satisfies

$$\begin{aligned} {\left\{ \begin{array}{ll} U^{m}_{zz}(z) -{c_0} U_z(z) + U(1 - U) = 0, \\ \lim _{z \rightarrow \infty }U(z) = 1, \\ U(z)> 0\quad \text { for } z > 0, \\ U(z) = 0\quad \text { for } z \le 0. \end{array}\right. } \end{aligned}$$

Consider a two parameters family of perturbations of the traveling wave solution *U* given by

3.2 $$\begin{aligned} V(z;\delta ,\zeta ) := (1 + \delta )U\big ({(1 + \delta )^{1 - \frac{m}{2}} \zeta z}\big ), \end{aligned}$$

where the parameters are bounded by $-\frac{1}{2}<\delta <\frac{1}{2}$ and ${\displaystyle \min _{x \in D}} \frac{1}{k^p(x)} \le \zeta \le {\displaystyle \max _{x \in D} } \frac{1}{k^p(x)}$. Then, the perturbed wave *V* satisfies

3.3 $$\begin{aligned} {\left\{ \begin{array}{ll} V^{m}_{zz} - c(\delta ,\zeta ) V_z + \zeta ^2V(1 + \delta - V) = 0 \ \text { for } z \in {\mathbb {R}}, \\ \lim _{z \rightarrow \infty }V(z; \delta , \zeta ) = 1 + \delta , \\ V(z; \delta , \zeta )> 0 \quad \text { for } z > 0, \\ V(z; \delta , \zeta ) = 0 \quad \text { for } z \le 0, \end{array}\right. } \end{aligned}$$

where

$$\begin{aligned} c(\delta ,\zeta ) = {c_0} (1 + \delta )^{\frac{m}{2}}\zeta . \end{aligned}$$

The perturbed waves are used to construct super- and sub-solutions in the proof of Theorem 2.2. The following lemma is for the properties of the perturbed wave *V*.

#### Lemma 3.4

The perturbed wave has the regularity $V\in C^2({\mathbb {R}}^+)\cap C({\mathbb {R}})$ and satisfies

3.4 $$\begin{aligned} V_{\zeta }&= \frac{z}{\zeta }V_{z},~~ V_{\zeta \zeta } = \left( \frac{z}{\zeta }\right) ^2 V_{zz},~~ V_{z\zeta } = \frac{1}{\zeta }V_{z} + \frac{z}{\zeta }V_{zz}, \end{aligned}$$

3.5 $$\begin{aligned} V_\delta&= \dfrac{V}{1 + \delta } + \dfrac{2 - m}{2(1 + \delta )} z V_z ,~~V_z> 0 \ \text { for } z >0. \end{aligned}$$

There exists a generic constant $C_V>0$ independent of $\delta$ and $\zeta$ such that, for $z > 0$,

3.6 $$\begin{aligned}&|c(0,\zeta ) - c(\delta ,\zeta )| \le C_V |\delta \zeta |, \end{aligned}$$

3.7 $$\begin{aligned}&0 < 1 + \delta - V \le C_V e^{- \beta z}, \end{aligned}$$

3.8 $$\begin{aligned}&V^{m}_{z} \le C_V V, \end{aligned}$$

3.9 $$\begin{aligned}&|z V_z| + |V^{m}_{zz}| + |z V^{m}_{zz}| \le C_V (V + V_z). \end{aligned}$$

#### Proof

The relations in (3.4) and (3.5) are directly obtained from the formula in (3.2). The estimate (3.6) is from definition of $c(\delta ,\zeta )$. We will show the rest for the case with $\delta =0$ only, and the general case is obtained by the continuous dependence of $c(\delta ,\zeta )$ and by taking the generic constant $C_V$ larger. Estimates in (3.7) and (3.8) can be found in Hilhorst et al. (2008). And in the same reference, we know that

$$\begin{aligned} |z V_z| \le C_V V \end{aligned}$$

for $z \ge 1$ and some positive constant $C_V$. Since

$$\begin{aligned} |z V_z| \le C_V V_z \end{aligned}$$

for $0< z < 1$ and $V,V_z\ge 0$, we obtain (3.9) for $|z V_z|$. By (3.3), we also obtain (3.9) for $|V_{zz}|$. By (3.7), we have

$$\begin{aligned} z(1 + \delta - V) \le C \end{aligned}$$

for a positive constant *C* since $z e^{- \beta z} \le \beta ^{-1}$ for $z > 0$. This implies that

$$\begin{aligned} |z V^m_{zz}| \le c |z V_z| + |z(1 + \delta - V)|V \le C_V(V + V_z). \end{aligned}$$

Also, since $|V^m_{zz}| \le c V_z + |\zeta ^2 (1 + \delta - V)|V$, the inequality also holds for $V^m_{zz}$ as well. $\square$

### Signed distance function

In this section, we consider the properties of the signed distance function $\tilde{d}(x,t)$ in a neighborhood of the surface that consists of the interfaces $\Gamma _t$ for $t\in [0,T]$ in $D\times [0,T]$ space. Denote

$$\begin{aligned} N(r,\tau ):= \{ (x,t) \in D \times [0,\tau ]: |\tilde{d}(x,t)| \le r \}. \end{aligned}$$

Then, clearly, $\cup _{t \in [0, \tau ]} \Gamma _t \times \{t\}\subset N(r,\tau )$ for all $r>0$ and $N(r,\tau )$ is a neighborhood of the surface that consists of interface $\Gamma _t$.

#### Lemma 3.5

There exist positive constants $d_0, C_d$ and *T* such that for all $(x,t) \in N(2d_0,T)$ the following holds;

3.10 $$\begin{aligned} \tilde{d} \in C^{2,1}(N(2d_0,T)) ,~~ |\nabla \tilde{d}| = 1 ,~~ |\tilde{d}_t(x,t) -{c_0} k^p(x,t)| \le C_d |\tilde{d}|. \end{aligned}$$

(Note that this implies that $\Gamma _t$ stays away from $\partial D$ by distance $2d_0$ for $t<T$.)

#### Proof

Under the assumption $\Gamma _0 \in C^{3 + \alpha }$ in (1.7), we can follow the construction of the interface motion equation in Chen and Reitich (1992) and rewrite the interface flow (2.4) in terms of a partial differential equation for $\tilde{d}$,

3.11 $$\begin{aligned} \tilde{d}_t(x,t) = c_0 k^p(y(x,t)), \end{aligned}$$

in a neighborhood of $\Gamma _t\times \{t\}$. In this formula, the heterogeneity in *k* is taken from $y(x,t) \in \Gamma _t$ that satisfies

$$\begin{aligned} dist(y(x,t), x) = | \tilde{d}(x,t)|. \end{aligned}$$

Such a point *y*(*x*, *t*) exists uniquely for $x\in N(2d_0,T)$ if the interface is smooth and $d_0$ is small.

$$\begin{aligned} y(x,t) = x - \tilde{d} \nabla \tilde{d} \end{aligned}$$

(see Gilbarg and Trudinger 2015, Section 14.6). Thus we obtain a partial differential equation for $\tilde{d}$,

3.12 $$\begin{aligned} \tilde{d}_t(x,t) = c_0 k^p \left( x - \tilde{d}(x,t) \nabla \tilde{d}(x,t) \right) . \end{aligned}$$

The conditions in (1.2) and (1.8), and Theorem 2 in Evans (2015, Section 3.2) imply the existence of the solution (3.12) in a set $N(2d_0,T)$ for a small constant $d_0>0$ with the regularity $\tilde{d} \in C^{2,1}(N(2d_0,T))$.

Note that, since the initial interface is smooth enough, $\Gamma _0 \in C^{3 + \alpha }$, we have $|\nabla \tilde{d}(x,0) | = 1$ for $x \in \{ x \in D: |\tilde{d}| \le 2d_0 \}$ by taking smaller $d_0$ if needed. Let $w(x,t):= |\nabla \tilde{d}|^2 - 1$. Using (3.12) we obtain

$$\begin{aligned} w_t&= 2 \nabla \tilde{d} \cdot \nabla \tilde{d}_t = \sum _{i=1}^N 2 \partial _{x_i} \tilde{d} ~ c_0 \nabla k^p \cdot (1^i - \partial _{x_i} \tilde{d} \nabla \tilde{d} - \tilde{d} \partial _{x_i} \nabla \tilde{d} )\\&= 2c_0 \sum _{i = 1}^N \partial _{x_i} \tilde{d} \partial _{x_i} k^p - 2 c_0 \nabla k^p \cdot \nabla \tilde{d} \sum _{i = 1}^N \partial _{x_i}\tilde{d}^2\\&\quad - 2 c_0 \tilde{d} \sum _{i = 1}^N \partial _{x_i} \tilde{d} \nabla k^p \cdot \partial _{x_i} \nabla \tilde{d}\\&= 2 c_0 \nabla {k^p} \cdot \nabla \tilde{d}~ (1 - |\nabla \tilde{d}|^2) - 2 c_0 \tilde{d} \sum _{i = 1}^N \sum _{j = 1}^N \partial _{x_i}\tilde{d} \partial _{x_j} k^p \partial _{x_i}\partial _{x_j} \tilde{d}\\&= - 2 c_0 \nabla {k^p} \cdot \nabla \tilde{d} ~w - c_0 \tilde{d} \sum _{i = 1}^N \sum _{j = 1}^N \partial _{x_j} k^p \partial _{x_j} (\partial _{x_i} \tilde{d}^2)\\&= - 2 c_0 \nabla {k^p} \cdot \nabla \tilde{d} ~w - c_0 \tilde{d} \sum _{j = 1}^N \partial _{x_j} k^p \partial _{x_j} |\nabla \tilde{d}|^2\\&= - 2 c_0 \nabla {k^p} \cdot \nabla \tilde{d} ~w -c_0 \tilde{d} \nabla k^p \cdot \nabla w, \end{aligned}$$

which is a first-order partial differential equation of *w*. By the characteristic technique with the initial value $w(x,0) = 0$, we obtain $w(x,t) = 0$ on $N(2d_0,T)$.

Using the relation (3.11), we have

$$\begin{aligned} |\tilde{d}_t(x,t) -{c_0} k^p(x)| = {c_0} |k^p(y(x,t)) - k^p(x)|. \end{aligned}$$

As $k^p$ is Lipschitz continuous, there exists a constant $C_d > 0$ such that

$$\begin{aligned} |\tilde{d}_t(x,t) -{c_0} k^p(x)| \le C_d ~ dist (y(x,t), x) = C_d |\tilde{d}(x,t)|, \end{aligned}$$

which is the third inequality in (3.10). $\square$

The constants $d_0$ and $C_d$ obtained in Lemma 3.5 are used in the rest of the paper. We construct a cut-off distance function using the constants. Let $h:{\mathbb {R}}\rightarrow {\mathbb {R}}$ be a monotone $C^2({\mathbb {R}})$ function that satisfies

$$\begin{aligned} h(s) ={\left\{ \begin{array}{ll} \ \ s &{} \text {if}~~ |s| \le d_0,\\ \ \ 2d_0 &{} \text {if}~~ s \ge 2d_0,\\ -2d_0 &{} \text {if}~~ s \le -2d_0. \end{array}\right. } \end{aligned}$$

The cut-off distance $d: D \times [0,T] \rightarrow {\mathbb {R}}$ is defined by

$$\begin{aligned} d(x,t) := h(\tilde{d}(x,t)). \end{aligned}$$

Then, by Lemma 3.5, we have, the cut-off distance function satisfies

3.13 $$\begin{aligned} |1 - |\nabla d|^2| \le C_d |d|,\quad |d_t -{c_0} k^p| \le C_d |d|,\quad |\nabla d| + |\Delta d| \le C_d \end{aligned}$$

by taking a larger $C_d > 0$ if needed.

## Generation of the interface

We prove Theorem 2.1 in this section. The uniform lower bound estimate of (2.1) is obtained by the comparison principle in Lemma 3.2 and the initial condition (1.6). The other estimates, including (2.2) and (2.3), are obtained by the comparison principle after constructing appropriate super- and sub-solutions. If $\eta _g \ge 1$, the estimate (2.2) is naturally obtained by (2.1). So, in this section, we assume $\eta _g \in (0,1)$. Note that the reaction term in (1.5) dominates the dynamics in the first stage of interface generation. Hence, it is natural to construct the super- and sub-solutions using the solution of the ordinary differential equation with the reaction terms. Let *Y* be the solution of

$$\begin{aligned} {\left\{ \begin{array}{ll} \frac{d}{d\tau }Y(\tau , \xi ; \delta )=Y(1-Y+ \delta ),\\ Y(0,\xi ; \delta ) = \xi , \end{array}\right. } \end{aligned}$$

where $|\delta | < \frac{1}{2}$. The spatial heterogeneity of the original problem (1.1) has been moved to the diffusion term as in (1.5). The obtained solution *Y* is similar to the solution in Alfaro et al. (2008), and the following properties of solution *Y* are taken from the paper:

### Lemma 4.1

Let $0< \eta _g < 1$. Then there exists a positive constant $C_Y = C_Y(\eta _g)$ and $\delta _0 = \delta _0(\eta _g)$ that satisfies the following estimates for all $|\delta | \le \delta _0$.

(i) For $\xi > 0$ and $\tau > 0$, we have $0 < Y_\xi (\tau , \xi ; \delta ) \le C_Y e^{(1 + \delta ) \tau }$.
(ii) For $\xi > 0$ and $\tau > 0$, we have $\left| \frac{Y_{\xi \xi }(\tau , \xi ; \delta )}{Y_\xi (\tau , \xi ;\delta )} \right| \le C_Y \left( e^{(1 + \delta ) \tau } - 1 \right)$.
(iii) For all $\tau > 0$ we have $Y(\tau , \xi ; \delta ) \le 1 + \delta$ if $\xi \le 1 + \delta$ and $Y(\tau , \xi ; \delta ) \le 0$ if $\xi \le 0$
(iv) There exists a positive constant $\varepsilon _Y$ such that for all $\varepsilon \in (0, \varepsilon _Y)$ we have $Y( |\ln \varepsilon |, \xi ; \delta ) \ge 1 + \delta - \eta _g$ if $\xi \ge C_Y \varepsilon , |\delta | \le \varepsilon$.

Now, we show the generation of the interface part.

### Proof of Theorem 2.1

For the proof, we choose a constant $0< \varepsilon _0 < \min \{ \varepsilon _Y, e^{-1},.\eta _g^{-1} \}$ so that

4.1 $$\begin{aligned} \varepsilon _0 \max _{x \in D} \dfrac{|\Delta (k(x))^m|}{k(x)} 2^{m- 1} \le \eta _g. \end{aligned}$$

We first prove (2.1). The inequality $0 \le u^\varepsilon$ is easily obtained since the function $w^-(x,t) \equiv 0$ is a sub-solution of $u^\varepsilon$. Let $w^+(x,t) \equiv 1 + \eta _\varepsilon$ where $\varepsilon \in (0, \varepsilon _0)$ and

4.2 $$\begin{aligned} \eta _\varepsilon = \varepsilon ^2 \max _{x \in D} \dfrac{|\Delta (k(x))^m|}{k(x)} 2^{m- 1}. \end{aligned}$$

Note that $\eta _\varepsilon \le \varepsilon _0 \eta _g < 1$ due to $\eta _g<1$ and the choice of $\varepsilon _0$. Direct computations of $\mathcal {L} w^+$ give

$$\begin{aligned} \mathcal {L}w^+&= - \dfrac{\varepsilon }{k}(1 + \eta _\varepsilon )^m\Delta k^m+ \dfrac{1}{\varepsilon } (1 + \eta _\varepsilon ) \eta _\varepsilon \\&= \dfrac{1 + \eta _\varepsilon }{\varepsilon } \left( \eta _\varepsilon - \dfrac{\Delta k^m}{k} \varepsilon ^2(1 + \eta _\varepsilon )^{m- 1} \right) \\&\ge \dfrac{1 + \eta _\varepsilon }{\varepsilon } \left( \eta _\varepsilon - \dfrac{\Delta k^m}{k} \varepsilon ^2 2^{m- 1} \right) \ge 0 . \end{aligned}$$

Thus, by the definition of $\eta _\varepsilon$, $w^+$ is a super solution, which implies (2.1).

Next, we prove (2.2) and (2.3). First, we extend the initial value $u_0$ to a $C^2$ function $\overline{u}_0: D \rightarrow {\mathbb {R}}$, which is available by Whitney extension theorem. Moreover, by condition (1.8) we can find a positive constant $\overline{d} < \min \{ d_0, 1 \}$ such that

$$\begin{aligned} \overline{u}_0 \le \overline{d} d(x,0) ~\text {if} ~ - \overline{d}< d(x,0) < 0. \end{aligned}$$

Then, we let $\sigma : D \rightarrow [0,1]$ be a smooth function satisfying

$$\begin{aligned} \sigma (x) \in {\left\{ \begin{array}{ll} 1 &{}~\text {if}~ d(x,0) > - \dfrac{\overline{d}}{2},\\ (0,1) &{}~\text {if}~ - \overline{d} < d(x,0) \le - \dfrac{\overline{d}}{2},\\ 0 &{}~\text {if}~ d(x,0) \le - \overline{d}. \end{array}\right. } \end{aligned}$$

With these functions in hand, we define $\tilde{u}_0: D \rightarrow {\mathbb {R}}$ as follows

$$\begin{aligned} \tilde{u}_0(x) := \sigma (x) \overline{u}_0 - (1 - \sigma (x)). \end{aligned}$$

Then we obtain

4.3 $$\begin{aligned} \tilde{u}_0 {\left\{ \begin{array}{ll}< \overline{d} d(x,0) &{}~\text {for} ~ - \overline{d} \le d(x,0) < 0,\\ = -1 &{}~\text {for} ~ - \overline{d} > d(x,0). \end{array}\right. } \end{aligned}$$

We construct functions $w^\pm (x,t)$ using this extended function as

$$\begin{aligned} w^\pm (x,t) =Y \left( \frac{t}{\varepsilon }, \max (\tilde{u}_0 \pm \varepsilon ^2 P(t),0); \pm \varepsilon \right) ,~ P(t) = K(e^{t/\varepsilon } - 1), \end{aligned}$$

where *K* is a positive constant. We take $\varepsilon _0$ smaller if needed so that $K\varepsilon _0\le 1/2$. Then, along with the facts that ${\tilde{u}}\le 1,\varepsilon \le 1$, we obtain $Y\le 2$. We will show that if *K* is chosen appropriately, we have

4.4 $$\begin{aligned} w^-(x,t) \le u^\varepsilon (x,t) \le w^+(x,t) \end{aligned}$$

for the solution $u^\varepsilon$ of (1.5). First, the initial values of the two functions are

$$\begin{aligned} w^\pm (x,0)=\max (\tilde{u}_0,0)=u_0(x), \end{aligned}$$

i.e., $w^\pm$ and *u* share the same initial value of the solution $u^\varepsilon$. Therefore, if we show $w^+$ is a super-solution and $w^-$ is a sub-solution, the claim (4.4) is obtained. We first show $w^+$ is a super-solution.

The conditions (*i*) and (*ii*) of Lemma 3.3 follows from the definitions of *Y* and $\tilde{u}_0$. Direct computations of $\mathcal {L}w^+$ on the support of $w^+$ give

$$\begin{aligned} \mathcal {L} w^+(x,t)&= \left( \frac{1}{\varepsilon } Y_\tau + K \varepsilon e^{t/\varepsilon } Y_\xi \right) - \frac{1}{\varepsilon }Y(1 - Y)\\&\quad - \frac{\varepsilon }{k} \left( (w^+)^m\Delta k^m+ 2 \nabla k^m\cdot \nabla (w^+)^m+ k^{m} \Delta (w^+)^{m} \right) \\&= K \varepsilon e^{t/\varepsilon } Y_\xi + Y - \frac{\varepsilon }{k} \left( Y^m\Delta k^m+ 2\nabla k^m\cdot ( mY^{m- 1} Y_\xi \nabla \tilde{u}_0 ) \right. \\&\quad \left. + k^m[ m(m- 1) Y^{m- 2} Y_\xi ^2 |\nabla \tilde{u}_0|^2 + mY^{m- 1} ( Y_{\xi \xi } |\nabla \tilde{u}_0|^2 + Y_\xi \Delta \tilde{u}_0 ) ] \right) \\&\ge \varepsilon Y_\xi \left( K e^{t/\varepsilon } - \frac{mY^{m- 2}}{k} \left( \left[ \left| \frac{Y_{\xi \xi }}{Y_\xi } \right| Y + (m- 1) Y_\xi \right] |\nabla \tilde{u}_0|^2 \right. \right. \\&\quad \left. \left. + Y|\Delta \tilde{u}_0| + Y|\nabla k^m\cdot \nabla \tilde{u}_0 | \right) \right) + Y \left( 1 - \varepsilon \frac{\Delta k^m}{k} Y^{m- 1} \right) . \end{aligned}$$

The last term in the inequality becomes non-negative since $Y\le 2$ and (4.1). Moreover, since we choose $\varepsilon _0 < e^{-1}$, for $0< \tau < t^\varepsilon$ we obtain

$$\begin{aligned} e^{(1 + \varepsilon )\tau / \varepsilon } \le C e^{\tau /\varepsilon } \end{aligned}$$

for a positive constant *C*. Then by conditions (1.2), (1.8) for the *k* and $u_0$ and Lemma 4.1, we can choose *K* large enough such that

$$\begin{aligned} \mathcal {L} w^+ \ge 0 \end{aligned}$$

for $0 < t \le \varepsilon |\ln \varepsilon |$. Therefore, $w^+$ is a super-solution. We can similarly show that $w^-$ is a sub-solution.

Next, we take $M_G \ge {\overline{d}}^{-1}(C_Y + K)$ and choose $\varepsilon _0$ small enough so that $(C_Y + K) \varepsilon _0 < 1$. For any point $x \in D$ satisfying $u_0(x) \ge M_G \varepsilon$ we have

$$\begin{aligned} \tilde{u}_0(x) - \varepsilon ^2 P(t^\varepsilon ) \ge M_G \varepsilon - K \varepsilon (1 - \varepsilon ) \ge C_Y \varepsilon , \end{aligned}$$

where the last inequality holds since $M_G \ge \overline{d}^{-1}(C_Y + K) \ge C_Y + K$ thanks to $\overline{d}\in (0,1)$. Thus, by Lemma 4.1 we obtain

$$\begin{aligned} u(x,t^\varepsilon )&\ge Y(|\ln \varepsilon |, \tilde{u}_0(x) - K \varepsilon (1 - \varepsilon ); - \varepsilon ) \ge 1 - \eta _g. \end{aligned}$$

And by (4.3), for any $x \in D$ satisfying $d(x,0) \le - M_G \varepsilon$ we have

$$\begin{aligned} \tilde{u}_0(x) + \varepsilon ^2 P(t^\varepsilon )&\le \max \{ \overline{d} d(x,0) , -1 \} + K\varepsilon (1 - \varepsilon )\\&\le \max \{ - \overline{d} M_G \varepsilon , -1 \} + K\varepsilon (1 - \varepsilon ) \le - C_Y \varepsilon \le 0. \end{aligned}$$

Thus, by Lemma 4.1 we obtain

$$\begin{aligned} u^\varepsilon (x,t^\varepsilon ) \le w^+(x,t^\varepsilon ) = 0. \end{aligned}$$

$$\square $$

## Propagation of the interface

We prove Theorem 2.2 in this section. If $\eta _p \ge 1$, the second estimate of (2.5) is trivial since $u^\varepsilon \ge 0$. Thus, in this section, we assume $\eta _p \in (0,1)$. We construct a pair of functions super- and sub-solutions $u^\pm$ as

5.1 $$\begin{aligned} u^\pm (x,t) = V \left( \frac{d(x,t) \pm \varepsilon p(t)}{\varepsilon }; \pm q(t), \frac{1}{k^p(x)} \right) , \end{aligned}$$

where

$$\begin{aligned} p(t) = - \dfrac{C_V C_k^p + 1}{\sigma } e^{- \sigma t/\varepsilon } + K_1 e^{Lt} ,~ q(t) = \dfrac{\eta _p}{2} e^{- \sigma t/\varepsilon } + K_2 \varepsilon . \end{aligned}$$

Here $C_k$ is the constant defined in (1.2), $C_V$ is the constant appearing in Lemma 3.4, $K_1, K_2, \sigma$ are some positive constants and $0< \eta _p < 1/4$. And we choose $\sigma \in (0, 1)$ small enough such that

5.2 $$\begin{aligned} \sigma \left( 1 + \dfrac{2 - m}{2} C_V \right)< 1 ,~~ \dfrac{2 - m}{4} \sigma C_V < 1. \end{aligned}$$

We make the following additional assumptions on $K_1, K_2, \varepsilon _0$ that

5.3 $$\begin{aligned} \left( \dfrac{C_V C_k^p + 1}{\sigma } + K_1 e^{LT}\right) \varepsilon _0< d_0 ,~ K_2\varepsilon _0 < \dfrac{\eta _p}{2}, \end{aligned}$$

which can be obtained by choosing $\varepsilon _0 > 0$ small enough. These imply that

5.4 $$\begin{aligned} \varepsilon _0 |p(t)| \le d_0 ,~~ q(t) \le \eta _p. \end{aligned}$$

Note that, if $m> 2$ we have $2 - m< 0$ which means that (5.2) holds for any $\sigma \in (0,1)$. We first prove that $u^\pm$ are a pair of sub- and super-solutions.

### Proposition 5.1

Let $K_1 > 1$. There exist positive constants $K_2, L, \varepsilon _0$ such that for any $0< \varepsilon < \varepsilon _0$ and $(x,t) \in D \times [0, T - t^\varepsilon ]$, $u^\pm$ defined by (5.1) satisfy

5.5 $$\begin{aligned} \mathcal {L}u^-(x,t) \le 0 \le \mathcal {L}u^+(x,t). \end{aligned}$$

### Proof

To show (5.5) we check the conditions $(i) - (iii)$ of Lemma 3.3 holds. The support of $u^\pm$ is equal to $\{ (x,t) \in D \times [0,T],~ d(x,t) \pm \varepsilon p(t) > 0 \}$, so its boundary in $D \times [0,T]$ is smooth by Lemma 3.5. Note that $w^\pm =0$ on $\partial D\times [0,T]$ since $d>-2d_0$ and $\Gamma _t$ is away from $\partial D$ by $3d_0$. With this, and by lemmas 3.5 and 3.4 we can see conditions (*i*) and (*ii*) hold. For (*iii*), we only show for $u^+$; one can use the same method for $u^-$ to show the condition (*iii*). For simplicity, we define ${z_d}= d(x,t) + \varepsilon p(t)$. A direct computation gives

$$\begin{aligned} u^+_t&=\left( \dfrac{d_t}{\varepsilon } + p'(t) \right) V_z + q'(t) V_\delta \\ \nabla V^m&= \dfrac{\nabla d}{\varepsilon }(V^m)_z + \nabla \dfrac{1}{k^p} (V^m)_\zeta = \left[ \dfrac{\nabla d}{\varepsilon } + \dfrac{{z_d}}{\varepsilon } k^p\nabla \frac{1}{k^p} \right] (V^m)_z\\ \Delta V^m&= \left| \dfrac{\nabla d}{\varepsilon }\right| ^2 (V^m)_{zz} + 2 \dfrac{\nabla d}{\varepsilon } \cdot \nabla \dfrac{1}{k^p} (V^m)_{z\zeta } + \left| \nabla \dfrac{1}{k^p}\right| ^2 (V^m)_{\zeta \zeta }\\&\quad + \nabla \cdot \left[ \dfrac{\nabla d}{\varepsilon } + \dfrac{{z_d}}{\varepsilon } k^p\nabla \frac{1}{k^p} \right] (V^m)_z\\&= \left[ \dfrac{\nabla d}{\varepsilon } + \dfrac{{z_d}}{\varepsilon } k^p\nabla \frac{1}{k^p} \right] ^2 (V^m)_{zz} + \nabla \cdot \left[ \dfrac{\nabla d}{\varepsilon } + \dfrac{{z_d}}{\varepsilon } k^p\nabla \frac{1}{k^p} \right] (V^m)_z\\&\quad + 2 k^p \dfrac{\nabla d}{\varepsilon } \cdot \nabla \dfrac{1}{k^p} (V^m)_z, \end{aligned}$$

where the equalities hold by (3.4). This implies that

$$\begin{aligned} \mathcal {L} u^+(x,t)&= \frac{d_t + \varepsilon p' }{\varepsilon } V_z + q' V_\delta \\&\quad - \frac{\varepsilon }{k} \left( k^m\Delta V^m+ 2 \nabla k^m\cdot \nabla V^m+ \Delta k^mV^m\right) - \frac{1}{\varepsilon } V (1 - V)\\&= \frac{d_t + \varepsilon p' }{\varepsilon } V_z + q' V_\delta \pm \frac{{c_\varepsilon }k^{2p}}{\varepsilon } V_z - \frac{1}{\varepsilon } V(1 - V) \pm \dfrac{q }{\varepsilon } V\\&\quad - \frac{\varepsilon }{k} \left( k^m\left[ \frac{\nabla d}{\varepsilon } + \frac{k^p{z_d}}{\varepsilon } \nabla \frac{1}{k^p} \right] ^2 V^m_{zz} \ \ + k^m\nabla \cdot \left[ \frac{\nabla d}{\varepsilon } + \frac{k^p {z_d}}{\varepsilon } \nabla \frac{1}{k^p} \right] V^m_z \right. \\&\quad \left. + 2 k^{p + m} \dfrac{\nabla d}{\varepsilon } \cdot \nabla \dfrac{1}{k^p} (V^m)_z + 2 \nabla k^m\cdot \left[ \frac{\nabla d}{\varepsilon } + \frac{k^p {z_d}}{\varepsilon } \nabla \frac{1}{k^p} \right] V^m_z + \Delta k^mV^m\right) \\&= \frac{d_t - {c_\varepsilon }k^{2p}}{\varepsilon } V_z + p' V_z + \frac{{c_\varepsilon }k^{2p}}{\varepsilon } V_z - \frac{1}{\varepsilon } V(1 + q - V) + \dfrac{q }{\varepsilon } V + q' V_\delta \\&\quad - \dfrac{\varepsilon }{k} k^m\dfrac{|\nabla d|^2}{\varepsilon ^2} V^m_{zz} - \dfrac{\varepsilon }{k} k^m\dfrac{k^p{z_d}}{\varepsilon } \nabla \dfrac{1}{k^p} \cdot \left[ 2 \dfrac{\nabla d}{\varepsilon } + \dfrac{k^p{z_d}}{\varepsilon } \nabla \dfrac{1}{k^p} \right] V^m_{zz}\\&\quad - \dfrac{\varepsilon }{k} \left( k^m\nabla \left[ \frac{\nabla d}{\varepsilon } + \frac{k^p {z_d}}{\varepsilon } \nabla \frac{1}{k^p} \right] V^m_z + 2k^{p + m} \nabla \frac{d}{\varepsilon } \cdot \nabla \frac{1}{k^p} V^m_z \right. \\&\quad \left. + 2 \nabla k^m\cdot \left[ \frac{\nabla d}{\varepsilon } + \frac{k^p {z_d}}{\varepsilon } \nabla \frac{1}{k^p} \right] V^m_z + \Delta k^mV^m\right) , \end{aligned}$$

where ${c_\varepsilon }:= c \left( q(t), \dfrac{1}{k^p} \right)$. Using (3.3) and the fact that $m- 1 = 2p$ we can rewrite $\mathcal {L}u^+ = E_1 + E_2 + E_3$, where

$$\begin{aligned} E_1&= p'(t) V_z + \dfrac{c_0 k^p - {c_\varepsilon }k^{2p}}{\varepsilon }V_z + q'(t) V_\delta + \dfrac{q(t)}{\varepsilon } V,\\ E_2&= \frac{d_t - c_0 k^p}{\varepsilon }V_z + \frac{1 - |\nabla d|^2}{\varepsilon } k^{2p} V^m_{zz} - \dfrac{\varepsilon }{k}\Delta k^mV^m,\\ E_3&= - k^{3p} \nabla \frac{1}{k^p} \cdot \left[ 2{\nabla d} + {k^p {z_d}} \nabla \frac{1}{k^p} \right] \frac{{z_d}}{\varepsilon } V^m_{zz} - 2k^{3p} \nabla {d} \cdot \nabla \frac{1}{k^p} V^m_z\\&\quad - \frac{1}{k} \left( k^m\nabla \left[ {\nabla d} + {k^p {z_d}} \nabla \frac{1}{k^p} \right] V^m_z + 2 \nabla k^m\cdot \left[ {\nabla d} + {k^p {z_d}} \nabla \frac{1}{k^p} \right] V^m_z \right) . \end{aligned}$$

(i) Estimates of $$E_1$$:

Since $K_1 > 1$, a direct computation gives

5.6 $$\begin{aligned} p'(t) V_z \ge \left( \dfrac{C_VC_k^p + 1}{\varepsilon } e^{-\sigma t/\varepsilon } + L e^{Lt} \right) V_z. \end{aligned}$$

By (3.5) we have

$$\begin{aligned} \dfrac{q(t)}{\varepsilon }V + q'(t) V_\delta =&K_2 V + \dfrac{\eta _p e^{- \sigma t/\varepsilon }}{2\varepsilon } V\\&- \sigma \dfrac{\eta _p e^{- \sigma t/\varepsilon }}{2\varepsilon } \left( \dfrac{V}{1 + q(t)} + \dfrac{2 - m}{2(1 + q(t))} \dfrac{{z_d}}{\varepsilon } V_z \right) . \end{aligned}$$

By (3.9) and (5.2) we obtain

5.7 $$\begin{aligned} \dfrac{q(t)}{\varepsilon }V + q'(t) V_\delta&\ge K_2 V \nonumber \\&\quad + \dfrac{\eta _p e^{- \sigma t/\varepsilon }}{2\varepsilon } \left( V - \sigma \left( V + \dfrac{2 - m}{2} C_V (V + V_z) \right) \right) \nonumber \\&\ge K_2 V - \dfrac{\eta _p e^{- \sigma t/\varepsilon }}{\varepsilon } \dfrac{2 - m}{4} \sigma C_V V_z \nonumber \\&\ge K_2 V - \dfrac{ e^{- \sigma t/\varepsilon }}{\varepsilon } V_z. \end{aligned}$$

And by (3.6) we have

5.8 $$\begin{aligned} \dfrac{V_z}{\varepsilon }(c_0 k^p - {c_\varepsilon }k^{2p})&\ge - C_V \dfrac{q(t)}{\varepsilon } k^p V_z \nonumber \\&\ge - C_V C_k^p \left( \dfrac{\eta _p}{2\varepsilon } e^{- \sigma t/\varepsilon } + K_2 \right) V_z \nonumber \\&\ge - C_V C_k^p \left( \dfrac{e^{- \sigma t/\varepsilon }}{\varepsilon } + K_2 \right) V_z, \end{aligned}$$

where the last inequality holds since $\eta \in (0,1)$. Thus the inequalities (5.6), (5.7) and (5.8) implies

5.9 $$\begin{aligned} E_1 \ge K_2 V + (L - C_1 K_2) V_z \end{aligned}$$

for a positive constant $C_1$.

(ii) Estimates of $E_2$:

By (3.13) we obtain

$$\begin{aligned} \dfrac{V_z}{\varepsilon }(d_t - c_0 k^{p}) + \frac{1 - |\nabla d|^2}{\varepsilon } k^{2p} V^m_{zz} \ge&- C_d \dfrac{|d|}{\varepsilon } (V_z + C_k^{2p} |V_{zz}^m|)\\ \ge&- C_d \dfrac{{z_d}}{\varepsilon } (V_z + C_k^{2p} |V_{zz}^m|)\\&- C_d p(t) (V_z + C_k^p |V_{zz}^m|). \end{aligned}$$

Thus by (3.9) we have following inequality

5.10 $$\begin{aligned} E_2 \ge - C_2(V + V_z) \end{aligned}$$

for a positive constant $C_2$.

(iii) Estimates of $E_3$:

Note that ${z_d}\le |d| + |p(t)| \le 2 d_0$ by (5.4). By (1.2), (3.9) and (3.13) we obtain

5.11 $$\begin{aligned} E_3 \ge - C_3 (V + V_z) \end{aligned}$$

for a positive constant $C_3$.

We now show $\mathcal {L}u^+ \ge 0$. By (5.9),(5.10) and (5.11) we have

$$\begin{aligned} \mathcal {L}u^+ \ge \left( K_2 - \tilde{C} \right) V + ( L - C_1 K_2 - \tilde{C}) V_z, \end{aligned}$$

where $\tilde{C} = C_2 + C_3$. Thus, by choosing *L* and $K_2$ large enough we have $\mathcal {L} u^+ \ge 0$. The other part for $\mathcal {L} u^- \le 0$ can be proved similarly. $\square$

### Proof of Theorem 2.2

The first inequality in (2.5) can be obtained by letting $w^- \equiv 0, w^+ \equiv 1 + \eta _\varepsilon$ as sub- and super- solutions, where $\eta _\varepsilon$ is a constant given in (4.2). We prove the rest of the results with $u^\pm$.

Fix $\eta _g \in (0, \eta _p)$, and let $\varepsilon _0, M_G$ be constants satisfying Theorem 2.1. In addition, we also assume $\eta _{\varepsilon _0}\le \eta _p/2$, which can be obtained by (4.2) and letting $\varepsilon _0$ small enough. By (1.8) we can find $C > 0$ such that

$$\begin{aligned} \text {if } d(x,0) \ge C \varepsilon&\text { then } u_0(x) \ge M_G \varepsilon ,\\ \text {if } d(x,0) \le - C \varepsilon&\text { then } u_0(x) = 0. \end{aligned}$$

With this, and by Theorem 2.1 we have

$$\begin{aligned} u^\varepsilon (x,t^\varepsilon ) \le H^+(x)&:= {\left\{ \begin{array}{ll} 1 + \eta _\varepsilon &{}~~ \text { if } d(x,0) \ge - C \varepsilon ,\\ 0 &{} ~~ \text { if } d(x,0)< - C \varepsilon , \end{array}\right. }\\ u^\varepsilon (x,t^\varepsilon ) \ge H^-(x)&:= {\left\{ \begin{array}{ll} 1 - \eta _g &{} ~~~ \text { if } d(x,0) \ge C \varepsilon ,\\ 0 &{} ~~~ \text { if } d(x,0) < C \varepsilon . \end{array}\right. } \end{aligned}$$

Equations (3.7) and (5.4) imply

$$\begin{aligned} V\left( z;q(0),\dfrac{1}{k^p(x)}\right) \ge 0 ,~ V\left( z;-q(0),\dfrac{1}{k^p(x)}\right) \le 1 - {\eta _p}/2 < 1 - \eta _g, \end{aligned}$$

for $x \in D, z \in {\mathbb {R}}$, where the last inequality holds by the choice of $\eta _g$. Moreover, for a fixed $K_1>0$ large enough and $x\in D$, we obtain

$$\begin{aligned} V\left( (-C + P(0)); q(0), \dfrac{1}{k^p} \right) \ge 1 + \eta _\varepsilon ,~ V\left( C -P(0); -q(0), \dfrac{1}{k^p} \right) = 0 \end{aligned}$$

since $\eta _\varepsilon \le \eta _p/2$, $P(0)=-\frac{C_VC^p_k+1}{\sigma }+K_1$ These inequalities and the monotonicity of *V* imply that

$$\begin{aligned} u^+(x,0) \ge H^+(x) ,~~ u^-(x,0) \le H^-(x). \end{aligned}$$

Thus, by Proposition 5.1 and Lemma 3.3 we have

5.12 $$\begin{aligned} u^-(x,t) \le u^\varepsilon (x,t + t^\varepsilon ) \le u^+(x,t) ~~ \text { for } ~~ x \in D, t \in [0,T - t^\varepsilon ]. \end{aligned}$$

By (3.7) and (5.3), we can choose $M_P > 0$ satisfying

$$\begin{aligned} V\left( (M_P - p(t)) ;~ - q(t), ~ \frac{1}{k^p} \right)&\ge 1 - \eta _p,\\ V\left( (- M_P + p(t));~ q(t), ~ \frac{1}{k^p} \right)&= 0 \end{aligned}$$

for any $(x,t) \in D \times [0, T - t^\varepsilon ]$. With this, and by (5.12) we have

$$\begin{aligned} u^\varepsilon (x,t + t^\varepsilon )\ge 1 - \eta _p\quad&\text {if }d(x,t) \ge M_P \varepsilon ,\\ u^\varepsilon (x,t + t^\varepsilon )= 0 \qquad&\text {if } d(x,t) \le - M_P \varepsilon . \end{aligned}$$

Therefore, the properties in (2.5) hold. $\square$

### Remark 1

Using the same sub- and super-solution $u^\pm$, we can also prove the convergence in (2.6). Indeed, by (3.7), we have

$$\begin{aligned} V\left( \beta ^{-1}|\ln \varepsilon |;-q(t), \dfrac{1}{k^p(x)} \right) \ge 1 - C_V \varepsilon - q(t). \end{aligned}$$

Moreover, we have $q(t) \le (\eta _p + K_2) \varepsilon$ for $t \ge t^\varepsilon = \varepsilon |\ln \varepsilon |$. Thus, we fix $C > 0$ large enough such that for a small enough $\varepsilon > 0$,

$$\begin{aligned} u^\varepsilon (x,t + t^\varepsilon ) \ge 1 - C \varepsilon ~~ \text {for}~~ d(x,t) \ge C \varepsilon |\ln \varepsilon | ,~ t \ge t^\varepsilon , \end{aligned}$$

where we used (5.12). Hence, (2.5) and the convergence of $t^\varepsilon \downarrow 0$ as $\varepsilon \downarrow 0$ imply (2.6).

## Numerical simulation

**Fig. 2:**
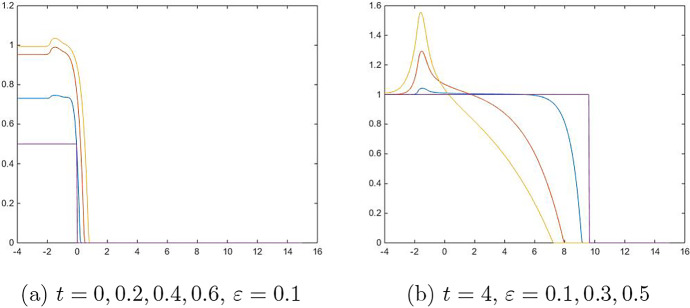
**a** Generation of interface. Equation (1.5) is solved with $\varepsilon = 0.1$ at $t=0,0.2,0.4, 0.6$(purple, blue, orange, yellow). **b** Propagation of interface. Equation (1.5) is solved for time $t=4$ with $\varepsilon = 0.1, 0.3, 0.5$ (blue, orange, yellow). The solution of interface motion (1.4), $\Gamma _{t=4}$, is given in purple (color figure online)

In this section, we compare solutions of the reaction–diffusion equation (1.5) and the interface motion equation (1.4) numerically. In these simulations, we take the exponent $m= 2$, where the minimum save speed for the homogeneous case $k=1$ is ${c_0} = 1$ (see Atkinson et al. 1981). In Fig. 2, numerical solutions are given when the space dimension is $N=1$, and the carrying capacity is

$$\begin{aligned} k(x)={\left\{ \begin{array}{ll} \ 0.5 &{} \text{ if } x<-1.5, \\ x+2 &{} \text{ if } x>-1.5. \end{array}\right. } \end{aligned}$$

Note that *k*(*x*) is piecewise harmonic and has a cusp at $x=-1.5$. The initial value is given by

$$\begin{aligned} u_0(x)=\frac{U_0(x)}{k(x)}={\left\{ \begin{array}{ll} 0.5 &{} \text{ if } x<0, \\ 0\ \ &{} \text{ otherwise }. \end{array}\right. } \end{aligned}$$

In Fig. 2a, numerical solutions for *u*(*x*, *t*) are given at four moments $t=0,0.2,0.4$, and 0.6 with fixed $\varepsilon =0.1$. This figure shows how the interface is developed. We can see that *u*(*x*, *t*) converges to 1 or 0 if *x* is away from the interface $x=0$ and the cusp $x=1.5$. In Fig. 2b, numerical solutions are given at time $t=4$ with three cases of $\varepsilon =0.1,0.3$ and 0.5. The step function in the figure (in the purple color) has been obtained after solving the interface motion equation (1.4). We can see that the solutions of the reaction–diffusion equation (1.5) converge to the step functions as $\varepsilon \rightarrow 0$.

**Fig. 3:**
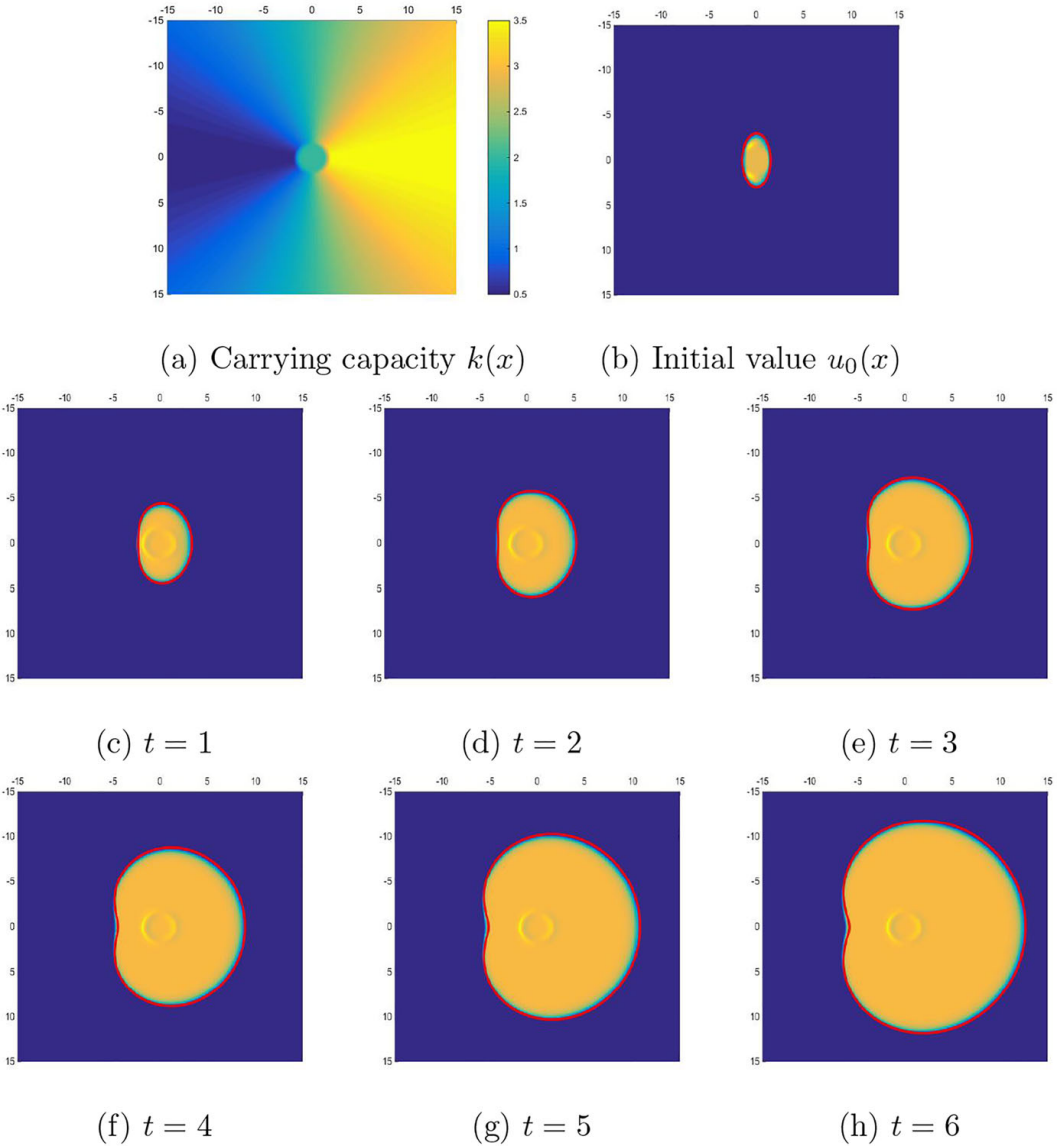
Snap shots of numerical the solutions of (1.5) with $m= 2$ and $\varepsilon = 0.1$. The closed curves in purple are obtained by solving the interface motion equation (1.4), which fits the transition layers of the simulation closely. We observe the development of a cavity on the interface (color figure online)

In Fig. 3, numerical solutions are compared in two space dimensions. The two-dimensional carrying capacity *k*(*x*) and the initial value $u_0(x)$ are given in Fig. 3a, b, respectively. More specifically, the heterogeneous carrying capacity *k*(*x*) is given in polar coordinates as

$$\begin{aligned} k(r,\theta ) = 2 + 1.5\cos \theta , \quad r\ge 2. \end{aligned}$$

For the region $r<2$, *k*(*x*) has been regularized to be a $C^2$ function as in Fig. 3a. We start with an initial value given in Fig. 3b. To observe the development of concavity more clearly, we let the initial value $u_0$ have a compact support of the ellipse shape.

Six snapshots of numerical solutions of (1.5) are given with time interval $\Delta t=1$ in Fig. 3c–h. The exponent power is still $m= 2$, and the singularity size is $\varepsilon = 0.1$ in these computations. The solutions of the interface motion equation (1.4) are marked in purple. As we can see from these snapshots, the boundary of the solution support is well approximated by the interface motion equation (1.4). The development of the concavity of the interface is clearly observed. As mentioned in the introduction, we no longer expect the solution support to be convex, even if it is initially. The convexity of the solution support may break due to the heterogeneity of the wave speed. We can observe a circular hump in the middle of the domain, which is due to the cusp of the carrying capacity *k*(*x*) at the same position. The same phenomenon was observed from Fig. 2b approximately at $x=-1.5$.

## Discussion

Dispersal dynamics and population growth are two key components that determine the propagation speed of an expanding biological species. Reaction–diffusion equations such as

$$\begin{aligned} U_t=d\Delta U^m+rU-bU^2, \end{aligned}$$

or equivalently,

7.1 $$\begin{aligned} U_t=d\Delta U^m+rU(1-U/k),\qquad k=r/b, \end{aligned}$$

are often used to model the phenomenon, where *d* is the diffusion coefficient, *r* is the growth rate, *b* is the competition coefficient, and *k* is the carrying capacity. If the coefficients are constant, we can easily see that the minimum wave speed is

7.2 $$\begin{aligned} c=c_0\sqrt{drk^{m-1}}=c_0\sqrt{dr^mb^{1-m}}, \end{aligned}$$

where $c_0$ is a proportionality constant. Its computation is simple. For example, if we set a new time variable $s=rt$, a new population unit $u=U/k$, and a new space variable $y=x/\sqrt{dk^{m-1}r^{-1}}$, then (7.1) is written as

$$\begin{aligned} u_s(y,s)=\Delta _y u^m+u(1-u). \end{aligned}$$

If $c_0$ is the minimum wave speed in these new variables, the wave speed in the original variables is the one in (7.2). The Fisher-KPP model is the case with the linear diffusion $m=1$, where the carrying capacity *k* does not affect the wave speed. Hence, one should consider a case $m\ne 1$ to see the effect of carrying capacity on the propagation speed, which is one of the main conclusions of the paper. In fact, it is often claimed that nonlinear diffusion with $m=2$, for example, fits the diffusion model for biological dispersal (e.g., see Hilhorst et al. 2008; Morishita 1971; Shigesada 1980).

Constant coefficients fit local phenomena of a small scale. For global phenomena of a large scale, environmental heterogeneity cannot be avoided, i.e., the three coefficients (*d*, *r*, *k*) are not constant anymore. In particular, the carrying capacity *k* and the diffusion coefficient *d* depend on the environment more than the intrinsic growth rate *r*. However, there is no traveling wave solution for such a heterogeneous case. To see the propagation speed in a heterogeneous environment, we have taken a hyperbolic scale problem

7.3 $$\begin{aligned} \varepsilon U_t=\varepsilon ^2\nabla \cdot \big (d(x)^{1-q}\nabla (d(x)^qU^m)\big )+r(x)U(1-U/k(x)), \end{aligned}$$

where $\varepsilon$ is the time and space scale of the biological level, and the solution gives a macroscopic level observation. For example, the scale of a rabbit’s habitat is 100 ms, and we want to observe the propagation of the rabbit population for the scale of 100 kms; the corresponding $\varepsilon$ is of order 0.001. Note that if the diffusivity *d*(*x*) is nonconstant, we should specify the diffusion law. The diffusion in (7.3) is Fick’s law if $q=0$, Chapman’s law if $q=1$, and Wereide’s law if $q=0.5$. However, we expect the wave speed of the $\varepsilon \rightarrow 0$ limit is independent of the exponent *q* and given by

7.4 $$\begin{aligned} c(x)=c_0\sqrt{d(x)r(x)k(x)^{m-1}}, \end{aligned}$$

which is identical to (7.2) locally. We have proved the convergence of the solution as $\varepsilon \rightarrow 0$ and the wave speed (7.4) when $m\ge 2$, *d* and *r* are constant. We expect that the cases with nonconstant *d*(*x*) can be handled similarly under similar conditions to *k*(*x*). However, the heterogeneity in the growth rate *r*(*x*) may behave very differently. The convergence proof will require a different approach and different hypotheses, such as monotonicity on *r* (see Evans and Souganidis 1989).

The hyperbolic scale in (7.3), which is obtained after rescaling parameters as $x\rightarrow \varepsilon x$ and $t\rightarrow \varepsilon t$, does not change the propagation speed of the solution. However, that does not mean the wave speed with a fixed $\varepsilon$ is the same as the one of the limit. The coefficient such as *k*(*x*) is not rescaled, so if $\varepsilon \rightarrow 0$, the surrounding environment appears to be expanding for a species that is shrinking. Therefore, it is not surprising that the formula (7.4) is identical to the homogeneous case (7.2) locally. On the other hand, if coefficients are also equally scaled, the problem can see the behavior of the solution at the origin only. Therefore, the next level is to understand the wave propagation speed with a fixed $\varepsilon >0$ and periodic coefficients.

A similar hyperbolic scale problem,

$$\begin{aligned} U_t(x,t) =\varepsilon \Delta (d(s)U)+\frac{1}{\varepsilon } U\Big (1-\frac{U}{k(x)}\Big ),\quad (x,t) \in D \times \mathbb {R}^+, \end{aligned}$$

has been considered in Hilhorst et al. (2023), which is a case of (7.3) with $r=m=q=1$. The special feature of the problem is that the motility *d* is an increasing function of the starvation measure $s=\frac{U(x,t)}{k(x)}$ (see Cho and Kim 2013). If the population density *U* is large and the carrying capacity *k* is small, the measure *s* is large, and so is the starvation measure *s*. In this problem, even if the carrying capacity is spatially heterogeneous, $k=k(x)$ and the diffusion is nonlinear, the propagation speed is homogeneous, and the corresponding interface motion equation is simply,

$$\begin{aligned} V_n = {\tilde{c}}_0, \end{aligned}$$

where ${\tilde{c}}_0$ is the traveling wave speed depending on the function $d(\cdot )$, but not on *k*(*x*). The starvation measure $s=\frac{U}{k}$ has a special property that makes the propagation speed unchanged when the motility is given by it. See Chang et al. (2024), Kim et al. (2014) for further use of the starvation-driven diffusion.
